# Contrast media extravasation injury: a prospective observational cohort study

**DOI:** 10.1186/s40001-023-01444-5

**Published:** 2023-10-25

**Authors:** Wanli Liu, Pinghu Wang, Hui Zhu, Hui Tang, Hongmei Guan, Xiaoying Wang, Chengxiang Wang, Yao Qiu, Lianxiang He

**Affiliations:** 1 Teaching and Research Section of Clinical Nursing, Xiangya Hospital, Central South University, Changsha, Hunan People’s Republic of China; 2 National Clinical Research Center for Geriatric Disorders, Xiangya Hospital, Central South University, Changsha, Hunan People’s Republic of China; 3 Breast Surgery Department, Hunan Provincial Maternal and Child Healthcare Hospital, Changsha, Hunan People’s Republic of China; 4 Xiangya Changde Hospital, Changde, Hunan People’s Republic of China

**Keywords:** Contrast media, Extravasation, Moderate extravasation injury, Risk factor

## Abstract

**Objective:**

To identify the risk factors for moderate and severe contrast media extravasation and provide effective guidance to reduce the degree of extravasation injuries.

**Methods:**

We observed 224 adult patients who underwent contrast media extravasation at Xiangya Hospital of Central South University, Hunan Provincial Maternal and Child Healthcare Hospital, and Xiangya Changde Hospital, Hunan Province between January 1, 2018 and December 31, 2022. Risk factors for moderate extravasation injuries were evaluated using univariate and multivariate logistic regression.

**Results:**

Among 224 patients, 0 (0%) had severe, 18 (8.0%) had moderate, and 206 (92.0%) had mild contrast media extravasation injury. Multivariate logistic regression analysis revealed malignant tumors (odds ratio [OR] = 6.992, 95% confidence interval [CI]: 1.674–29.208), Iohexol (OR = 9.343, 95% CI 1.280–68.214), large-volume (> 50 mL) extravasation (OR = 5.773, 95% CI 1.350‒24.695), and injection site (back of the hand) (OR = 13.491, 95% CI 3.056–59.560) as independent risk factors for moderate injury.

**Conclusion:**

Risk factors for moderate contrast media extravasation injury are malignant tumors, iohexol, large-volume (> 50 mL) extravasation, and back-of-the-hand injection. Analysis of these risk factors can help reduce the degree of injury after extravasation.

**Clinical relevance statement:**

High-risk patients with extravasation support should choose the appropriate contrast media type, avoiding back-of-the-hand injections. We recommend that patients with cancer be implanted with a high-pressure resistant central venous catheter and receive effective measures to timely detect and reduce extravasation.

## Key points

1. Severe extravasation injury in contrast media is rare.
2. The incidence of moderate extravasation injury in contrast media was 8.0% (18 / 224).
3. Malignant tumor, Iohexol, large-volume(> 50 mL) extravasation, and back of the hand injection affect the degree of injury after contrast media extravasation.

## Introduction

With the rapid development of multi-detector computed tomography, there are approximately 76 million examinations in the world each year, of which 50% of patients need to use contrast media (CM) intravenously [1], which is on the increase. CM are mainly used for qualitative and differential diagnosis of pathological changes based on the absorption difference between normal and diseased tissues; however, they need to be injected into the human body through the vein with instantaneous high pressure and a rapid method. In addition to its special physical and chemical properties (such as high concentration, high osmotic pressure, and high viscosity), various complications can occur after injection, of which CM extravasation is the main complication [2], with an incidence rate of 0.1‒1.2% [3–10]. The diagnosis of CM extravasation is mainly based on a comprehensive clinical judgment of the time and symptoms of extravasation [11, 12].

Some studies have shown that extravasation causes pain, swelling, blisters, secondary wound infection, tissue adhesion, and even serious consequences such as compartment syndrome and amputation [13–15], which increase the patient's pain, prolong the hospital stay, increase medical costs, and affect the patient's disease diagnosis, treatment, and rescue, ultimately affecting the patient's quality of life.

To reduce the incidence of CM extravasation, many research teams have made significant efforts, but due to high-pressure injection and the physicochemical properties of CM drugs, patient age, underlying diseases, and other reasons, CM extravasation is difficult to completely avoid [16]. When CM extravasation cannot be avoided, we hope to take some measures to minimize the harm caused by extravasation. Through a literature review, we found that there have been few studies on the damage caused by CM extravasation, and some studies have been reported in the form of individual cases. Some studies have mentioned the grading of extravasation damage, but there is a lack of continuous follow-up after extravasation. There are also studies that cannot further analyze and discuss moderate and severe CM extravasation due to insufficient sample size.

Therefore, this study aims to analyze the risk factors for moderate and severe injuries caused by CM extravasation by following up on cases of CM extravasation in the radiology departments of three hospitals over a period of 5 years. Taking preventive measures in advance to reduce extravasation damage for patients who may be at high risk of extravasation is needed to avoid serious harm caused by extravasation.

## Methods

### Patients and study design

The study was conducted following the guiding principles of the Declaration of Helsinki and approved by the Xiangya Hospital Ethics Committee of Central South University (202110200). Because the data were analyzed anonymously, the ethics committee waived the need for informed consent.

This observational cohort study involved 224 adult patients who underwent CM extravasation at Xiangya Hospital of Central South University, Hunan Provincial Maternal and Child Healthcare Hospital, and Xiangya Changde Hospital, Hunan Province, from January 1, 2018, to December 31, 2022. The inclusion criteria were as follows: (l) patients who underwent CM extravasation between January 1, 2018 and December 31, 2022; and (2) patients older than 18 years of age. The exclusion criteria were as follows: (1) patients who did not cooperate with the follow-up; and (2) patients who died during the follow-up.

### CM injection

Three hospitals used CM, including iodixanol, iohexol, ioversol, iomeprol, iopromide, iopamidol, etc., and before injection, all CM were externally heated to a target temperature of 37 °C to reduce viscosity. For enhanced-computed tomography, if the patient had a high-pressure resistant central venous catheter (the maximum tolerable pressure was 300 psi), the radiology nurse drew blood back to confirm that the catheter was in the vein and used 20 mL of normal saline to confirm its patency before use. Central venous catheters labeled with high-pressure resistant labels can be used for high-pressure injection of iodine containing contrast agents. In addition, the radiology nurse selected the 18G‒22G [17] high-pressure resistant peripheral short catheter (the maximum tolerable pressure was 350 psi) according to the proposed injection speed and the patient's vascular conditions.

Before the intravenous injection of CM, the radiology nurse again conducted routine tests on all intravenous routes by manually injecting normal saline to determine whether the catheter was unobstructed and to confirm that the catheter was located in the vein. The patients were instructed to raise their hands to inform the medical staff if injection site pain or other discomforts suddenly occurred during the computed tomography high-pressure injection of CM*.*

### Record after CM extravasation

In the case of CM extravasation, the patient was initially evaluated by a nurse in the computed tomography department. The outpatient patients were followed up by the intravenous treatment nurse in the computed tomography department, whereas the inpatients were handed over by the computed tomography department nurse to the patient's original ward nurse, and the ward nurse conducted the subsequent follow-ups. We evaluated the degree of CM extravasation and filled in a special record. The records contained the following information: the patient's basic information, injection speed, estimated amount of CM extravasation, venous access sites, related symptoms and signs (such as pain, swelling, skin redness, sensory changes, and other symptoms), and selection for CM extravasation management.

### Assessment of the degree of CM extravasation injury

To evaluate the degree of the CM extravasation injury, three hospitals used the evaluation form to conduct a dynamic evaluation after extravasation. Mild extravasation injury was defined as pain, swelling, and/or mild erythema. Moderate extravasation injury was defined as moderate or severe erythema, blistering, evident pain, swelling, inflammation, or lesions requiring additional treatment, and all symptoms resolved within 2 weeks. Severe extravasation injury was defined as serious adverse effects, such as cyanosis and tissue necrosis, or symptoms lasting more than 2 weeks, such as persistent pain and edema, difficulty in locomotion, and cases requiring surgery [18] (Table 1).

**Table 1:** Drug extravasation evaluation form

Classification	Description	Symptom chart
Mild extravasation damage	Pain, swelling, and/or mild erythema	
Moderate extravasation damage	Moderate or severe erythema, blistering or evident pain or swelling, inflammation or lesions requiring additional treatment. All symptoms resolved within 2 weeks	
Severe extravasation damage	Serious adverse effects; cyanosis, tissue necrosis, or symptoms lasting more than 2 weeks, such as persistent pain and edema, difficulty in locomotion, and cases requiring surgery	

After the occurrence of CM extravasation, outpatients were observed in the injection room of the radiology department for 2 h. If the injection site did not show any signs of blistering or evident pain, they could leave the hospital accompanied by their family members and receive daily follow-up. If the symptoms worsen or become uncontrollable after leaving the hospital, the hospital's intravenous treatment nurses and wound nurses will intervene in a timely manner for treatment. Inpatients were observed in the original inpatient department and were not discharged within 24 h after extravasation. If the symptoms did not worsen or become uncontrollable after 24 h, they can be discharged and undergo daily follow-up.

If the symptoms of severe injury persisted for more than 3 days and could not be relieved completely or had a tendency to deteriorate into acute compartment syndrome and necrosis of the overlying skin [19–21], the patient was transferred to the wound center or surgical ward.

### Data collection

We prospectively collected information on the sex, age, body mass index (BMI), type of CM, and whether the patients had a malignant tumor, diabetes, venous thrombosis, hypoalbuminemia, pulmonary infection, multi-site angiography, and/or injection rate ≥ 3 mL/s, injection site, injection side, and extravasation volume. Extravasation volume is an approximate value, determined by estimating the amount of CM entering the body in the image and examining the remaining amount of CM in the medicine bottle. Multi-site angiography means that patients have multiple body sites imaged via different contrast injection rates through the same intravenous access site. The patients were followed up until the extravasation injury healed, permanent injury, or death.

### Statistical analysis

Descriptive statistics were used to evaluate the study population and the incidence of moderate CM extravasation injuries. All data were analyzed using SPSS for Windows (version 22.0; SPSS Inc., Chicago, IL, USA). The measurement data were expressed as the mean ± standard deviation.

The statistical description of enumeration data was expressed in terms of frequency and percentage. Univariate and multivariate logistic regression analyses were used to estimate the odds ratios (ORs) and 95% confidence intervals (CIs) of the association between all potential risk factors and moderate CM extravasation injury. All results with *p* < 0.05 were statistically significant. The corresponding measure of effect or association and confidence interval are reported along with the significance tests.

## Results 

### Basic information of CM extravasation patients

Table 2 shows that of the 231 patients assessed for eligibility, 6 patients were lost to follow-up and 1 patient died, resulting in the failure to track the outcome of CM extravasation. Finally, 224 adult patients with complete data and who had CM extravasation were included in the analysis. Among these, 0 (0%) had severe injuries, 18 (8.0%) had moderate injuries, and 206 (92.0%) had mild CM extravasation injuries. The symptom response of the patients peaked 48 h after injection. The average moderate extravasation injury healing time is 7‒14 days, and the average mild extravasation injury healing time is 2‒4 days.

**Table 2:** Comparison of variables between patients with and without moderate extravasation injury

Variable	Moderate extravasation injury	Mild extravasation injury
	*n* = 18 %	*n* = 206 %
Sex		
Men	7 (38.89)	102 (49.51)
Women	11 (61.11)	104 (50.49)
Age (years)		
Age ≤ 50	2 (11.11)	52 (25.24)
50 < age ≤ 60	8 (44.44)	60 (29.13)
60 < age < 70	1 (5.56)	66 (32.04)
Age ≥ 70	7 (38.89)	28 (13.59)
BMI		
> 25	5 (27.78)	49 (12.79)
≤ 25	13 (72.22)	157 (76.21)
Malignant tumor		
Yes	13 (72.22)	76 (36.89)
No	5 (27.78)	130 (63.11)
Diabetes		
Yes	6 (33.33)	33 (16.02)
No	12 (66.67)	173 (83.98)
Venous thrombosis		
Yes	2 (11.11)	17 (8.25)
No	16 (88.89)	189 (91.75)
Hypoalbuminemia		
Yes	2 (11.11)	29 (14.08)
No	16 (88.89)	177 (85.92)
Pulmonary infection		
Yes	7 (38.89)	48 (23.30)
No	11 (61.11)	158 (76.70)
Type of contrast media		
Iodixanol	3 (16.67)	84 (40.78)
Iohexol	8 (44.44)	27 (13.10)
Ioversol	5 (27.78)	63 (30.58)
Others^a^	2 (11.11)	32 (15.54)
Multi-site angiography		
≤ 2	5 (27.78)	79 (38.35)
> 2	13 (72.22)	127 (61.65)
Injection rate (mL/s)		
Injection rate < 3	12 (66.67)	100 (48.54)
Injection rate ≥ 3	6 (33.33)	106 (51.46)
Large volume (≥ 50 mL) extravasation		
Yes	11 (61.11)	61 (29.61)
No	7 (38.89)	145 (70.39)
Insertion side		
Left	11 (61.11)	52 (25.24)
Right	7 (38.89)	154 (74.76)
Injection site		
Forearm and elbow fossa	4 (22.22)	153 (74.27)
Back of the hand	14 (77.78)	48 (23.30)
Neck	0 (0.00)	5 (2.43)

Others^a^ contrast media include iomeprol, iopromide, iopamidol, etc.

All patients received conservative treatment, including raising the venipuncture point and cold compress; local application of dexamethasone and compound aescin gel. (This product is a polypill. Its components are aescin and diethylamine salicylic acid.) No patient received additional treatment intervention with conservative treatment. Except for three cases of skin pigmentation, there were no further complications or sequelae of CM extravasation during follow-up.

### Univariate analyses of risk factors for moderate extravasation injury

Table 3 shows the results of univariate and multivariate analyses of moderate extravasation injury risk factors. Age ≥ 70 years, malignant tumors, large-volume (> 50 mL) extravasation, insertion on the left side, and injection site (back of the hand) were important variables in the univariate analysis.

**Table 3:** Univariate analyses for the moderate extravasation injury

Variable	Odds ratio (95% confidence interval)	*P-value*
Univariate analysis		
Sex (reference: female)	0.649 (0.242‒1.740)	0.390
Age (reference: ≤ 50 years)		
50 < age ≤ 60	3.467 (0.705‒17.057)	0.126
60 < age < 70	0.394 (0.035‒4.465)	0.452
Age ≥ 70	6.500 (1.264‒33.416)	0.025
BMI ≥ 25	1.232 (0.418–3.629)	0.705
Malignant tumor	4.447 (1.526‒12.960)	0.006
Diabetes	2.621 (0.919‒7.478)	0.072
Venous thrombosis	1.390 (0.295‒6.557)	0.678
Hypoalbuminemia	0.763 (0.167‒3.494)	0.727
Pulmonary infection	2.095 (0.770‒5.701)	0.148
Multi-site angiography	0.618 (0.212‒1.801)	0.378
Injection rate ≥ 3 mL/s	0.472 (0.171‒1.305)	0.148
Type of contrast media (reference: Iodixanol)		
Iohexol	8.296 (2.054–33.507)	0.013
Ioversol	1.750 (0.279–10.963)	0.550
Others	2.222 (0.512–9.647)	0.286
Large volume (> 50 mL) extravasation	3.735 (1.383‒10.090)	0.009
Insertion left side	4.654 (1.715‒12.630)	0.003
Injection site (reference: Forearm and elbow fossa)		
Back of the hand	11.156 (3.506‒35.500)	< 0.001
Neck	0.000 (0.000‒0.000)	0.999

Others^a^ contrast media include iomeprol, iopromide, iopamidol, etc.

### Multivariate analyses of risk factors for moderate extravasation injury

Multivariate logistic regression analysis revealed malignant tumors (OR = 6.992, 95% CI 1.674–29.208), iohexol (OR = 9.343, 95% CI 1.280–68.214), large-volume (> 50 mL) extravasation (OR = 5.773, 95% CI 1.350‒24.695), and injection site (back of the hand) (OR = 13.491, 95% CI 3.056–59.560) as independent risk factors for moderate injuries (Table 4).

**Table 4:** Multivariate analyses for the moderate extravasation injury

Variable	Odds ratio (95% confidence interval)	*P-value*
Multivariate analysis		
Age (reference: ≤ 50 years)		
50 < age ≤ 60	1.528 (0.226‒10.336)	0.664
60 < age < 70	0.177 (0.011‒2.730)	0.215
Age ≥ 70	2.278 (0.249‒20.823)	0.466
Malignant tumor	6.992 (1.674–29.208)	0.008
Type of contrast media (reference: Iodixanol)		
Iohexol	9.343 (1.280–68.214)	0.028
Ioversol	1.419 (0.137–14.682)	0.769
Others^a^	2.273 (0.343–15.068)	0.395
Large volume (> 50 mL) extravasation	5.773 (1.350‒24.695)	0.018
Insertion left side	3.508 (0.817‒15.059)	0.091
Injection site (reference: Forearm and elbow fossa)		
Back of the hand	13.491 (3.056–59.560)	0.001
Neck	0.000 (0.000‒0.000)	0.999

Others^a^ contrast media include Iomeprol, Iopromide, Iopamidol, etc.

## Discussion

Studies have shown that the mechanisms of different degrees of injury caused by CM extravasation mainly include the following: 1. cytotoxicity: in vitro studies have shown that CM can cause morphological degeneration of human umbilical vein endothelial cells [22] and induce apoptosis of endothelial cells [23]. 2. Mechanical compression: the degree of mechanical compression is directly related to the amount of extravasation. High-dose CM extravasation can cause severe rupture of external venous tissues, especially when high-pressure injections and injection sites are difficult to monitor [4]. 3. Osmosis: hyperosmolar CM extravasated in the subcutaneous tissue increases local tissue osmotic pressure. The dehydration of the vascular endothelial cell network causes local platelet aggregation, which causes inflammatory changes by increasing capillary permeability, leukocyte infiltration, and releasing other inflammatory chemokines, such as prostaglandin E1 and E2 [5].

Analysis of registered data across the United States by Dykes et al. showed that mild, moderate, and severe incidences of injuries caused by CM extravasation were 94.6%, 4.7%, and 0.8%, respectively [2]. In contrast, we had no cases of severe injury; instead, potentially deteriorating cases were controlled at the moderate injury stage. This may be related to the CM-type selection of the three hospitals in the study. Since the development of CM, they have gone through a development process from ionic to non-ionic, from high-osmolality to low-osmolality to isotonic: (1) high-osmolality CM have osmotic pressures as high as 5–7 times that of plasma; Due to the relatively high number of adverse reactions, it is currently rarely used; (2) low-osmolality CM are named after their significantly reduced osmotic pressure compared to ionic high-osmolality CM. They include two types: non-ionic monomers and ionic dimers, with osmotic pressure twice that of plasma; (3) after further reducing the osmotic pressure of the low permeability CM, an isotonic contrast agent was developed, but due to its current high price, it has not been widely used.

The CM used in the radiology departments of the three hospitals was the non-ionic type, which is the dominant type with less necrosis, edema, and hemorrhage compared with ionic CM [12]. In the classification of non-ionic CM, we found that the use of iohexol for injection is one of the risk factors for moderate CM injury. The osmotic pressure of iohexol is 830 mOsm/kg H_2_O, which is higher than those of iodixanol (290 mOsm/kg H_2_O) and ioversol (710 mOsm/kg H_2_O). Plasma osmotic pressure is composed of oncotic pressure (macromolecular blood proteins), and crystal osmotic pressure is composed of inorganic salts, glucose, and other small molecules. The normal value is 280–310 mOsm/kg H_2_O, and blood proteins generally cannot penetrate the capillary wall; therefore, although the plasma oncotic pressure is low, it plays an important role in the water balance inside and outside the blood vessels. Once the hypertonic fluid is extravasated, the plasma osmotic pressure and tissue osmotic pressure gradually increase, and patients are more likely to experience local skin redness, pain, damage, necrosis, dehydrated vascular endothelial cells, local platelet aggregation, leukocyte infiltration, and inflammatory damage. In addition, the higher viscosity (11.4 mPa∙s) and lower hydrophilicity (the octanol water partition coefficient: 708) of iohexol may result in more severe damage than ioversol and iodixanol.

Because massive extravasation leads to a mechanical compression effect [24, 25], the amount of extravasation is directly proportional to the damage caused by extravasation [2, 7, 10, 23, 24], and our research also confirms this. To reduce the amount of CM leakage, we suggest that during the high-pressure injection of CM, the nurse closely observes the patient's reaction and changes in the pressure curve on the high-pressure display screen through the intercom and video system in the observation window. If the pressure line suddenly increases or exceeds the pressure value of 250 psi (1 psi = 6.895 kPa), the injection of CM should be stopped immediately. The patient should be asked through the intercom to see if there is swelling at the injection site, and measures should be taken promptly for symptomatic treatment. It is recommended to use an extravasation detection accessory (EDA) to assist in monitoring the occurrence of CM extravasation. The main principle of EDA is to use abnormal soft tissue at the insertion site to signal the operator to stop injection when CM extravasation occurs, and automatically terminate injection before a large amount of extravasation occurs. In recent years, various leakage monitoring and alarm technologies have developed rapidly. The research results of Dykes et al. [2] showed that the incidence and total amount of CM extravasation in patients who used extravasation monitoring were significantly lower than those who did not use it (*P* < 0.05).

Tumors are also risk factors for moderate extravasation injuries. Some studies have shown that chemotherapy status does not affect the incidence of drug extravasation [26]. However, it may stimulate the veins and make them more vulnerable to subsequent treatment [27]. In addition, the disease state of patients with tumors and the decline of immunity after chemotherapy caused the body to react more heavily after drug extravasation, and the injury was more difficult to heal. With the development of medicine, most patients with tumors undergoing chemotherapy have been implanted with a central venous catheter, including the CVC, PICC, and Port. We recommend that patients with high-pressure injections choose high-pressure resistant central venous catheters at the beginning. In our study, we were surprised to find out that patients with high-pressure-resistant central venous catheters did not have CM extravasation. High-pressure resistant central venous catheters are usually rated at 300 psi with flow rates up to 10 mL/s [28], which can meet the speed and pressure requirements of intravenous contrast-enhanced CT.

The degree of CM extravasation injury is also related to the injection site of the peripheral short catheter [18]. Compared with the loose subcutaneous layer, the involvement of the tight subfascial compartment causes more injury [29]. Therefore, extravasation injury usually indicates worse consequences at the injection site with a thinner subcutaneous plane, such as the back of the hand and wrist [25], which is consistent with the 13.491 (*p* = 0.001) times of moderate extravasation in the back of the hand compared with the high-pressure injection in the forearm and elbow fossa in this study. Therefore, we suggest that when CM is injected intravenously through a peripheral venous catheter, the catheter should be preferentially placed in the venous with thick, straight, and good elasticity at the cubital fossa, including the median cubital vein, cephalic vein, and basilic vein, and should be avoided from puncturing at the hands, wrists, feet, and ankles as much as possible. If the vein at the elbow fossa is not suitable for puncture, the forearm vein can be chosen. Meanwhile, avoid repeated punctures of the same vein.

Our study prospectively collected information on patients with CM extravasation. The results provide a reference for reducing the degree of injury after CM extravasation. Our findings support early risk assessment for patients requiring angiography and the selection of high-pressure-resistant central venous catheters for patients with malignant tumors. To take effective measures to reduce CM extravasation, patients at high risk of extravasation support should avoid back-of-the-hand injections and iohexol to reduce moderate and severe CM extravasation injuries.

### Limitations

This study has some limitations. Due to the fact that all patients with CM extravasation have undergone raising the venture point and cold compress, local application of dexamethasone and compound aescin gel management, there is no control group (patients with CM extravasation are not treated in this way). Therefore, the effectiveness and efficiency of this management technology could not be investigated. In addition, since the data were from three centers, there may be differences in equipment or technician operations.

### Conclusions

The risk factors for moderate extravasation injury in CM are malignant tumors, iohexol, large-volume (> 50 mL) extravasation, and back-of-the-hand injection. The analysis of these risk factors can help reduce the degree of injury after CM extravasation.

## Data Availability

The datasets used and/or analyzed during the current study are available from the corresponding author on reasonable request.

## Abbreviations

BMI: Body mass index
CM: Contrast media
ORs: Odds ratios
Cis: Confidence intervals
